# Association between Handgrip Strength, Mobility, Leg Strength, Flexibility, and Postural Balance in Older Adults under Long-Term Care Facilities

**DOI:** 10.1155/2019/1042834

**Published:** 2019-09-23

**Authors:** Agnieszka Wiśniowska-Szurlej, Agnieszka Ćwirlej-Sozańska, Natalia Wołoszyn, Bernard Sozański, Anna Wilmowska-Pietruszyńska

**Affiliations:** ^1^Department of Medicine, Institute of Physiotherapy, University of Rzeszow, Rzeszow, Poland; ^2^Center for Innovative Research in Medical and Natural Sciences, University of Rzeszow, Rzeszow, Poland; ^3^Department of Medicine, Lazarski University, Warsaw, Poland

## Abstract

**Introduction:**

Low muscle strength is common and important in geriatric syndromes including frailty and sarcopenia. The epidemiology of grip strength of older people under long-term care facilities has been little explored.

**Purpose:**

The aim of this study was to assess handgrip strength of older women and men covered by institutional care and to analyse the associations between HGS and mobility, leg strength, flexibility, and postural balance.

**Materials and Methods:**

This is a cross-sectional study carried out at care homes in southeastern Poland. After considering the inclusion criteria, 209 older people aged 65 to 85 were included in the study. Sociodemographic data were collected, and tests of muscular strength, mobility, flexibility, and postural balance were carried out by the use of the stabilometric platform CQ Stab 2P.

**Results:**

The average handgrip strength in the study group amounted to 19.8 kg, including 14.8 kg in women and 25.9 kg in men. Low grip strength was found in 67.83% women and 52.13% men in institutional care. A negative correlation between handgrip strength (HGS) and the Timed Up and Go (TUG) test was demonstrated, both with and without cognitive task and strength of lower limbs. Gait speed and dynamic balance were positively correlated with HGS. A negative correlation was found between the total length of the centre of pressure (COP) path, the length of the COP path in the lateral-medial direction, and the sway area delimited by the COP and HGS for the dominant hand. Speaking of women, gait speed was most strongly associated with HGS, while among men, it was upper limb flexibility.

**Conclusion:**

Regardless of gender, HGS is associated with mobility, strength of the lower limbs, and dynamic balance. By means of simple tools, early diagnosis will facilitate the planning of appropriate interventions in order to prevent disability and mortality in long-term care facilities.

## 1. Introduction

Aging is associated with progressive loss of muscle mass with a simultaneous increase in fat mass [1]. A decrease in skeletal muscle mass takes place at the rate of 3–8% for a decade and begins after 30 years of age [2]. Its loss is also accompanied by a significant decrease in muscle strength amounting to more than 15% per decade [3].

Loss of muscle strength is a key indicator for many geriatric syndromes, including weakness syndrome, sarcopenia, mobility impairments, and falls [4]. Weaker grip strength is tightly associated with multiple morbidities [5] and poorer self-rated health [6]. Epidemiological studies have shown that it is an important indicator of the risk of cognitive impairment, dementia, and depression in older people [7, 8]. It was also found that age, gender, body mass index (BMI), and nutritional state correlate with HGS [9, 10].

Muscle strength is an important determinant of healthy aging [11]. Low handgrip strength (HGS) is a strong predictor of mobility impairment, both in women and men [12]. The decrease in the grip strength associated with aging reduces the independence of the older people, leading to the need to use family support or caregivers [13]. It may impair manual dexterity of upper limbs, as well as affect the ability to maintain postural balance and gait independence [14]. It is used to predict disability, morbidity, and mortality in the future [15]. Early detection of low muscle strength can help identify people at risk of significant mobility restrictions and increased bedtime [16].

The European Working Group on Sarcopenia in Older People (EWGSOP) recognizes that strength is a better measurement than muscle mass in predicting loss of independence or need for long-term care placement. EWGSOP recommends using a range of tools in the assessment of older people, which includes handgrip strength, chair stand, gait speed, TUG test, and balance assessment. For individual measurements, cutoff points for diagnostic variables for people at risk of weakness are specified [17]. However, there are few data assessing the correlation between HGS and other feasible measures of mobility, leg strength, flexibility, and postural balance in the population of older people covered by institutional care.

Functional disability, in the face of demographic changes, is a challenge for public health. Due to the fact that the average grip strength varies depending on the geographic regions of the world, the extension of reference values among older women and men receiving institutional care in Poland is important for clinical practice [18]. The aim of the study was to assess the strength of the handgrip and identify factors associated with it among older women and men in long-term care facilities.

## 2. Materials and Methods

### 2.1. Study Setting

It is a cross-sectional study carried out in randomly selected 9 residential care homes in the southeast of Poland.

### 2.2. Participants

**Figure 1:**
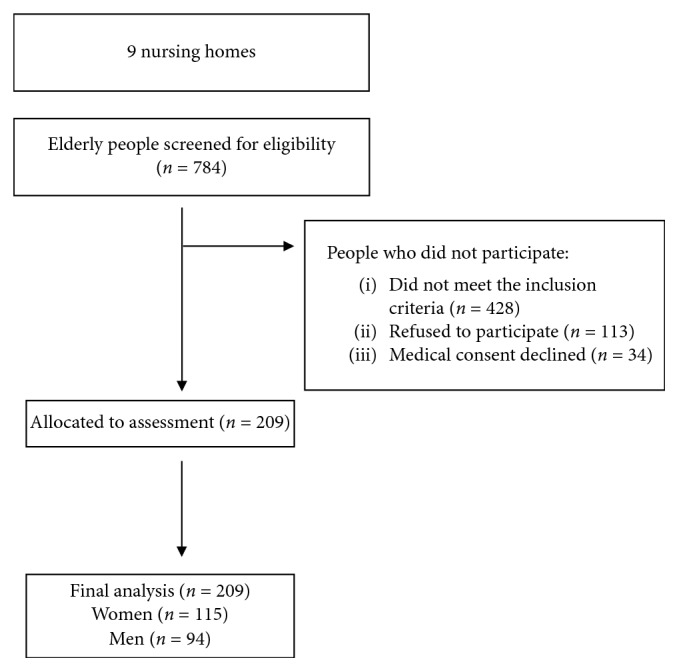
Flow diagram of participants through the study.

The study involved older people who lead a sedentary lifestyle staying in residential long-term care facilities in southeastern Poland. The criteria for inclusion in the study were age from 65 to 85 years, a normal cognitive status or a mild impairment in the field of orientation and memory examined by Mini-Mental State Examination (MMSE) from 30 to 19 points, no or moderate depression in the Geriatric Depression Scale (GDS) below 11 points, a level of physical performance enabling the subject to take a standing position on the stabilometric platform, and physically inactive—performing activities in a sitting position, such as reading and watching TV, for a minimum of 4 hours a day/6-7 days a week. Exclusion criteria were vestibular and neurological disorders, dizziness, taking drugs significantly affecting the body's balance, injuries of the lower limbs during the last 6 months, paresis or deformities in the upper limbs, and severe systemic diseases. Regarding 784 residents of care homes and after considering inclusion criteria and receiving written consent of the residents to participate in the project, 209 people were included in the study. A flow diagram shows participant selection and dropout (Figure 1).

### 2.3. Procedure

The study was conducted by a research team in two stages. On the first day, sociodemographic data were collected and anthropometric measurements were carried out, whereas on the second day, functional tests were carried out.

Data regarding age, sex, education, and marital status were collected on the basis of records kept by care homes and an interview with the researched people. Data considering chronic diseases were collected from medical records kept by doctors in care homes. The diseases were categorized and divided into 4 main groups: cardiovascular, neurological, musculoskeletal, and urinary tract diseases. Information on the number of taken medications was also collected.

Body height measurements were recorded to the nearest centimetre by the use of a stadiometer, and body weight was measured to the nearest kilogram by the use of a digital weigh scale. Body mass index (BMI) was calculated as weight in kg divided by height in meter squared and classified according to World Health Organization categories [19].

The assessment of cognitive abilities was carried out using the MMSE [20] and mood assessment using GDS [21]. The preferred form of spending free time was determined by asking the question of how much time the participant spent in sitting position

#### 2.3.1. Handgrip Strength (HGS)

The assessment was carried out by the use of a hand dynamometer (JAMAR PLUS + Digital Hand Dynamometer, Patterson Medical) calibrated by the manufacturer. The measurement was performed in a sitting position, on a chair without armrests, with the feet of the examined person resting flat on the floor, arms set along the torso, the elbow flexed at 90 degrees, the forearm in a neutral position, and the wrist in 0 degrees to 30 degrees extension following the recommendations of the American Society of Hand Therapists [22]. The subject was instructed to clench the hand maximally and hold for 6 seconds. The procedure was repeated three times for the dominant hand, with a one-minute rest between the tests. The average of three measurements (in kilograms) was recorded. Normal and low grip strength values were established according to the criteria proposed by the EWGSOP [17].

#### 2.3.2. Timed Up and Go (TUG)

Mobility assessment of the subject was made on the basis of a specific sequence of movements: getting up from the chair (height 41 cm with back support), walking a distance of 3 meters, rotating 180 degrees, covering the distance back to the chair, and sitting again [23]. The test was carried out in three attempts. The sample with the shortest time (s) was selected for the assessment.

#### 2.3.3. Timed Up and Go Cognitive (TUG cog)

The test was performed the same way as in the TUG assessment, but while the test was being performed, the cognitive task was added. The older person was asked to subtract constantly the number 3 starting from the number indicated by the tester.

#### 2.3.4. Gait Speed

Assessment of the walking speed was carried out using a 10-meter corridor test. The test assessed the time taken by an elderly person to cover a distance of 10 meters. The test was carried out in two attempts. The first attempt was getting to know the test, and the second (proper) consisted in fast (but safe) gait reaching the destination [24]. Walking speed was calculated by dividing the distance by the time needed to cover the distance (m/s).

#### 2.3.5. Lower Limb Strength

Lower limb strength was assessed by the use of the chair stand test [25]. The elderly people were ordered to stand up from the chair 5 times and sit on it without the help of upper limbs, at the fastest possible pace. The time needed to perform the test (s) was measured.

#### 2.3.6. Upper Body Flexibility

Upper body flexibility was assessed by the use of the back stretch test [26]. The study was carried out in the standing position. The elderly were asked to stretch one hand up and over the shoulder and reach down the back and the other hand behind and reach up the back, with the intention of meeting the hands in the middle of the back, between the shoulder blades. The distance between the tips of the middle fingers of both hands was measured in centimetres: if the fingertips just barely touched, the score was zero; the distance of overlapping fingertips was recorded as a plus (+) score; the distance between the tips of the middle fingers was recorded as a minus (‐) score if they did not touch.

#### 2.3.7. Lower Body Flexibility

Lower body flexibility was assessed by the use of the chair sit and reach test [27]. The participants were asked to sit on the edge of a chair. One leg stayed flat on the floor, and the other leg was extended as straight as possible in front of the hip with the heel placed on the floor and with the ankle flexed at approximately 90 degrees. Participants were asked to stretch out the arms with overlapping hands and slowly bend forward at the hip joint reaching as far forward as possible toward or past the toes. The assistant measured the distance from the middle fingertips to the top of the toes in centimetres: if the fingertips touched the toes, then the score was zero; if they did not touch at this point, the distance was recorded as a minus (‐) score; if they overlapped, the distance was recorded as a plus (+) score.

#### 2.3.8. Postural Stability

Assessment of the postural stability was performed by the use of a two-plate stability platform CQ Stab 2P (CQ Elektronik System, Poland). Each of the platform plates had 3 force sensors that determined the displacement of the centre of pressure on the support plane. During the measurements, the values describing the static balance were recorded. The platform plates were placed parallel, 2 m from the wall of the room where there was a marker for fixing eyesight during the test with open eyes. Each time before the measurements were taken, the device was calibrated. The test consisted of a 30-second sample performed with eyes open and eyes closed. The subjects were instructed to remove shoes and take a free-standing position on the platform plates with their arms set along the trunk [28]. The higher the value of the parameters recorded by the platform, the more the COP displacement was on the support plane [29].

The following parameters were used in the analysis:

- SP: total path length measures on the *XY* axes in mm
- SPAP: statokinesiogram path length measured in the *Y* axis direction in mm
- SPML: statokinesiogram path length measured in the *X* axis direction in mm
- MA: mean COP displacement (radius) in mm
- MAAP: mean COP displacement from point 0 in the *Y* axisdirection in mm
- MAML: mean COP displacement from point 0 in the *X* axis direction in mm
- MaxAP: maximal COP displacement from point 0 in the *Y* axis direction in mm
- MaxML: maximal COP displacement from point 0 in the *X* axis direction in mm.

### 2.4. Ethical Approval and Informed Consent

In accordance with the Declaration of Helsinki, the participants were informed about the aim and the course of the study and gave their informed consent to take part. Due to representative nature of the results obtained in the study, they allowed us to gain knowledge about a large community by examining its representation. The study design was approved by the Bioethical Committee of the University of Rzeszow (No. 6/06/2015).

### 2.5. Statistical Analysis

The collected data were analysed with the use of TIBCO Software Inc. (2017) Statistica (data analysis software system), version 13. The preliminary analysis used the measurements of descriptive statistics. Pearson's correlation coefficient was calculated in order to assess two-dimensional correlation between examined parameters. Regression with dependent variables was used to determine the relationship between HGS and parameters assessing leg strength, flexibility, and body balance after adjusting for age, sex, and BMI. Statistical significance was set at *p* < 0.05.

## 3. Results

The study included 115 women (55.02%) and 94 men (44.98%). The average age of the entire study group was 74.6 years, while the average age of women was significantly higher than men. This is in line with the general population trend in Poland. Most of the participants were widows or widowers (40.67%). Over 60% of people had primary or vocational education. Generally, the study group was dominated by people with normal body weight (39.71%) and obesity (39.23%), with women in majority having normal weight, while men were mostly obese. Over 40% of studied people had normal cognitive status. Most patients did not have depression (66.51%). Cardiovascular and musculoskeletal diseases dominated in the study group. The average number of drugs taken in the study group was on average 4 items. The data on the state of health did not differentiate the researched women and men. The average strength of handgrip in the studied group was 19.8 kg, and for women, it was 14.8 kg and for men 25.9 kg. Over 60% of the researched people were characterized by reduced handgrip strength (including 67.83% women and 52.13% men). There were differences between sexes for age, body mass, height, marital status, handgrip, mobility, gait speed, right upper limb flexibility, and postural balance variables describing the mean and maximal COP displacement in the anteroposterior direction. Characteristics of participants are shown in Table 1.

Considering received data negative correlation was found between HGS and age, TUG test, both with and without a cognitive task and the lower limb strength. BMI, gait speed, and dynamic balance were positively correlated with HGS. Futhermore, there was no correlation between upper and lower body flexibility with HGS for the dominant hand. A negative correlation was identified between the total path length COP, the length of the COP path in the lateral-medial direction, and the sway area delimited by the COP and HGS for the dominant hand. In addition, among women, gait speed and lower limb flexibility were positively correlated with HGS, whereas lower limb strength and the total path length COP and the length of the COP path in the lateral-medial direction were correlated negatively. Among men, upper right hand flexibility and left lower limb flexibility were positively associated with HGS, while age, lower limb strength, total path length COP, and the length of the COP path in the anterior-posterior and lateral-medial direction were negatively correlated (Table 2).

After adjusting for age, sex, and BMI and gender interaction, a relationship between handgrip strength and mobility has been demonstrated with and without cognitive task, as well as gait speed, lower limb strength, and dynamic body balance. No effect of gender interaction with HGS has been shown on these dependent variables. Significantly higher values of the parameters of the total COP path length and lateral-medial COP path length under visual control have been found in men than in women. The relationship between HGS and mobility, leg strength, and postural balance after age adjustment, sex, and BMI is shown in Table 3.

## 4. Discussion

The research showed that regarding women, gait speed was the most strongly associated with HGS, while in men, it was the flexibility of the upper limb, gait speed, strength of the lower limbs, and dynamic body balance with reference to older people living in residential care homes.

HGS is a good indicator determining the risk of disability and mortality. Therefore, there is an increasing interest in its assessment in clinical settings. In our own research, it was shown that the average strength of the handgrip in the study group amounted to 19.8 kg, including 14.8 kg for women and 25.9 kg for men. Al Snih et al. showed that 42% of older women with a handgrip strength of less than 14 kg and 38% men with handgrip strength less than 22 kg died within 5 years [30]. A recent study containing normative data from the FNIH Sarcopenia Project regarding the strength of handgrip and further life course indicated that the limit for the occurrence of the weakness syndrome is HGS for women under 16 kg and for men under 27 kg [31]. The own research showed that 67.83% of women and 52.13% of men had reduced muscle strength. This percentage is much higher than the observations carried out in the older population living in community [32]. The data considering HGS in the general population were carried out in many countries. However, there are no data on the incidence of reduced muscle strength in older people in long-term care facilities [33].

As a result of the analyses, a statistically significant negative correlation between age and HGS was observed in the entire study group. Similar results were obtained by Silva Nde et al. [34]. For each year, over 60 years of age, there is a decrease in the mean handgrip strength by 0.1 kg [33]. A longitudinal study among the Danish older population indicated that men were losing HGS faster than women, but they remained independent in their daily activities for longer [35].

A weak positive correlation between HGS and BMI was observed in the examined group of people. The results obtained in the study suggest that the occurrence of overweight or obesity may be a factor determining greater muscle strength. The results of previous studies were varied. Underweight was associated with low HGS and obesity with a high parameter [6, 36]. Wearing et al. did not show any relations between BMI and HGS [37]. The results discrepancy may be due to the lack of optimal BMI reference values for the older population [38].

The results of our own research presented that the mobility (TUG) with and without the cognitive task, regardless of sex, was negatively correlated with HGS. This means that people with lower handgrip strength were characterized by a longer duration of the task. Porta et al. also showed correlations between TUG and muscle strength [39]. The authors indicted that all TUG parameters were significantly correlated with HGS. Lino et al. also observed that cognitive impairment was significantly associated with weaker handgrip strength [40]. The TUG test reflects physiological changes occurring with age [41]. A slow decrease in reaction time, reduction of nerve conduction velocity, and reduction of sensorimotor responses may lead to balance disorders, postural abnormalities, and then mobility and walking speed restrictions [42].

The measure of gait speed and grip strength is accurate and specific [43]. As a result of the performed research, it was shown that the gait speed was positively correlated with HGS. Muscle strength affects the variability of gait speed [44]. With regards to older people, there is a decrease in gait parameters. A decrease in self-efficiency, balance disorders, and fear of falls influences the phenomenon of a more cautious and slower gait pattern, which transfers to its speed [45].

The results of our own research showed a correlation between dynamic body balance and HGS. Fujita et al. also showed a significantly lower handgrip strength in the case of greater balance disorders [46]. In order to maintain vertical balance, the centre of body mass should remain within the limits of the quadrilateral support defined by the foot contour. Moving the COP beyond the limits of foot support causes loss of balance, which is interfaced with the motor response [47]. A decrease in muscle strength, delay in muscle activation, and slower reaction time affect greater balance disorders [48]. Body balance is considered to be a crucial element of many everyday activities, starting from maintaining a calm position to more complex activities, such as walking during a conversation or a change of the walking direction [49]. Detection of existing balance disorders is important for preventing falls and planning improvement strategies.

The research showed that HGS correlated with the flexibility of the lower limbs in women and the right upper and lower left limbs in men. Muscle flexibility is important because its limitations have a large impact on the movement performance [50]. Silva Nde et al. obtained different results and pointed out that the reasons for the lack of a linear correlation between flexibility and muscular strength in older people are not clear and recommend conducting further analyses [34].

Our own research indicated the existence of a strong relationship between HGS and the strength of the lower limbs in both genders. The regression result shows that the strength of the lower limbs depends on HGS and age. Fragala et al. also observed a significant correlation between grip strength and leg extension strength [51]. HGS correlates with the knee extension strength of both the ipsilateral and contralateral sides, which favours the use of handgrip strength as a measure of global strength [52, 53].

The measurement of screening test to the needs of clinical practice can be a measure of handgrip strength with the help of a dynamometer. The implementation requires minimal staff training, the duration of the test is about 2 minutes, and the cost of measuring equipment is affordable for most medical facilities. Lower HGS was significantly associated with weaker muscular strength of the lower limbs, gait speed, and balance, and thus, it is a predictive measure to determine the general functional status of an older person. Quick identification of people with weakness syndrome gives the opportunity to optimize health management and implement appropriate rehabilitation exercises.

Our research has some limitations. First of all, no data considering the diet of older people were collected as well as body mass composition was not assessed, which could be a better indicator than BMI. Secondly, due to cross-sectional data, the study does not permit considerations on causality.

## 5. Conclusion

Low grip strength was found in 67.83% women and 52.13% men in institutional care. HGS, regardless of gender, is associated with mobility, strength of the lower limbs, and dynamic balance. HGS assessment can be a simple, fast, and inexpensive way to assess the prevalence of mobility limitations and functional performance. Early diagnosis will facilitate the planning and application of appropriate interventions in order to prevent disability and mortality in long-term care facilities.

## Figures and Tables

**Table 1:** Characteristics of the studied group.

	Women	Men	Total	*p* value
Age (years)	76.2 (7.8)	72.7 (8.1)	74.6 (8.1)	0.002
Body mass (kg)	65.9 (15.6)	78.5 (15.0)	71.6 (16.5)	<0.001
Height (cm)	158.4 (8.2)	171.3 (7.6)	164.2 (10.2)	<0.001
BMI (kg/m^2^)				0.275
Underweight	3 (2.61)	4 (4.26)	7 (3.35)	
Normal body weight	51 (44.35)	32 (34.04)	83 (39.71)	
Overweight	16 (13.91)	21 (22.34)	37 (17.71)	
Obesity	45 (39.13)	37 (39.36)	82 (39.23)	
Marital status				<0.001
Married	3 (2.61)	17 (18.09)	20 (9.57)	
Widow/widower	56 (48.70)	29 (30.85)	85 (40.67)	
Divorced	16 (13.91)	17 (18.09)	33 (15.79)	
Single	40 (34.78)	31 (32.98)	71 (33.97)	
Education				0.084
Basic	35 (30.43)	41 (43.62)	76 (36.36)	
Vocational	35 (30.43)	30 (31.91)	65 (31.10)	
Secondary	42 (36.53)	20 (21.28)	62 (29.67)	
Higher	3 (2.61)	3 (3.19)	6 (2.87)	
Chronic disease				
Cardiovascular	100 (89.96)	81 (86.17)	181 (86.60)	0.868
Musculoskeletal	76 (66.09)	52 (55.32)	128 (61.24)	0.112
Neurological	25 (21.74)	19 (20.21)	44 (21.05)	0.787
Pulmonary	61 (53.04)	43 (45.74)	104 (49.76)	0.294
Urinary system	20 (17.29)	16 (17.02)	36 (17.22)	0.944
GDS				0.458
No depression	79 (68.70)	60 (63.83)	139 (66.51)	
Moderate depression	36 (31.30)	34 (36.17)	70 (33.49)	
MMSE				0.710
No cognitive impairment	45 (39.13)	39 (41.49)	84 (40.19)	
Cognitive impairment without dementia	34 (29.57)	23 (24.47)	57 (27.27)	
Mild dementia	36 (31.30)	32 (34.04)	68 (32.54)	
Number of drugs	3.9 (1.7)	4.1 (1.6)	4.0 (1.7)	0.513
Number of falls	0.8 (1.1)	0.6 (1.0)	0.7 (1.0)	0.180
Strength				
Handgrip dominant (kg)	14.8 (6.4)	25.9 (9.8)	19.8 (9.8)	<0.001
Cutoff points handgrip strength dominant (kg)	78 (67.83)	49 (52.13)	127 (60.77)	
Chair stand (s)	22.83 (9.83)	20.36 (8.82)	21.72 (9.45)	0.083
Mobility				
TUG (s)	20.44 (9.77)	17.88 (9.17)	19.28 (9.56)	0.009
TUG cog (s)	25.04 (12.28)	21.44 (10.14)	23.42 (11.48)	0.019
Gait speed (m/s)	0.60 (0.25)	0.69 (0.27)	0.64 (0.26)	0.007
Flexibility				
Upper limb flexibility_R_ (cm)	–27.68 (14.10)	–33.30 (14.30)	–30.20 (14.42)	0.002
Upper limb flexibility_L_ (cm)	–31.92 (14.96)	–32.27 (14.76)	–32.07 (14.83)	0.868
Lower limb flexibility_R_ (cm)	–10.56 (12.87)	–12.51 (14.05)	–11.43 (13.41)	0.286
Lower limb flexibility_L_ (cm)	–11.04 (13.23)	–13.53 (13.35)	–12.15 (13.30)	0.116
Body balance				
BERG	34 (13)	36 (13)	35 (13)	0.214
Postural balance (eyes open)				
SP (mm)	485.00 (330.78)	567.48 (498.83)	522.10 (415.83)	0.132
SPAP (mm)	385.27 (293.035)	455.01 (409.59)	416.64 (351.11)	0.083
SPML (mm)	211.79 (138.64)	243.06 (251.78)	225.86 (197.80)	0.278
MA (mm)	5.46 (3.14)	6.29 (4.02)	5.84 (3.58)	0.039
MAAP (mm)	3.82 (1.92)	4.85 (3.32)	4.28 (2.69)	0.004
MAML (mm)	2.99 (2.72)	3.00 (2.31)	3.00 (2.54)	0.456
MaxAP (mm)	16.59 (11.08)	19.69 (15.68)	17.98 (13.40	0.030
MaxML (mm)	14.59 (17.80)	14.25 (16.91)	14.43 (17.36)	0.816
Postural balance (eyes closed)				
SP (mm)	559.85 (525.71)	558.53 (445.76)	559.25 (490.16)	0.904
SPAP (mm)	474.64 (472.58)	470.44 (402.63)	472.75 (441.51)	0.982
SPML (mm)	210.88 (172.25)	210.78 (166.38)	210.83 (169.22)	0.418
MA (mm)	4.77 (2.73)	5.13 (2.88)	4.93 (2.79)	0.403
MAAP (mm)	3.91 (2.42)	4.10 (2.34)	3.99 (2.37)	0.462
MAML (mm)	1.97 (1.29)	2.23 (1.69)	2.08 (1.48)	0.470
MaxAP (mm)	17.37 (12.43)	17.03 (11.14)	17.21 (11.84)	0.872
MaxML (mm)	7.77 (5.79)	10.54 (15.13)	9.01 (11.06)	0.565

*N*, number; SD, standard deviation; BMI, body mass index; GDS, Geriatric Depression Scale; MMSE, Mini-Mental State Examination; TUG, Timed Up and Go; TUG cog, Timed Up and Go cognitive; SP, total path length; SPAP, statokinesiogram path length; SPML, statokinesiogram path length; MA: mean COP displacement; MAAP, mean COP displacement from point 0 in the *Y* direction; MAML, mean COP displacement from point 0 in the *X* direction; MaxAP, maximal COP displacement from point 0 in the *Y* direction; MaxML, maximal COP displacement from point 0 in the *X* direction.

**Table 2:** Correlation between HGS and different variables among older adults by sex.

	Handgrip (kg)					
	Women	*p* value	Men	*p* value	Total	*p* value
Age (years)	–0.18	0.053	–0.11	0.029	–0.23	0.001
BMI (kg/m^2^)	0.15	0.102	0.14	0.165	0.16	0.022
Mobility						
TUG (s)	–0.17	0.073	–0.11	0.295	–0.18	0.008
TUG cog (s)	–0.15	0.119	–0.07	0.532	–0.17	0.014
Gait speed (m/s)	0.30	0.001	0.10	0.319	0.24	<0.001
Strength						
Chair stand (s)	–0.27	0.004	–0.24	0.018	–0.27	<0.001
Flexibility						
Upper limb flexibility_R_ (cm)	0.16	0.098	0.42	<0.001	0.13	0.060
Upper limb flexibility_L_ (cm)	–0.04	0.703	–0.18	0.185	–0.08	0.245
Lower limb flexibility_R_ (cm)	0.24	0.010	0.15	0.137	0.11	0.102
Lower limb flexibility_L_ (cm)	0.20	0.035	0.25	0.012	0.13	0.655
Body balance						
BERG	0.18	0.051	0.09	0.397	0.15	0.030
Postural balance (eyes open)						
SP (mm)	–0.19	0.046	–0.30	0.003	–0.16	0.023
SPAP (mm)	–0.16	0.080	–0.27	0.010	–0.13	0.058
SPML (mm)	–0.22	0.017	–0.30	0.003	–0.18	0.008
MA (mm)	–0.14	0.129	–0.14	0.184	–0.05	0.483
MAAP (mm)	–0.10	0.282	–0.12	0.237	0.01	0.831
MAML (mm)	–0.15	0.111	–0.14	0.174	–0.11	0.103
MaxAP (mm)	–0.05	0.576	–0.05	0.615	0.02	0.752
MaxML (mm)	–0.12	0.213	–0.13	0.207	–0.11	0.128
Postural balance (eyes closed)						
SP (mm)	–0.09	0.326	0.17	0.111	0.03	0.653
SPAP (mm)	–0.09	0.334	0.18	0.089	0.04	0.616
SPML (mm)	–0.10	0.304	0.09	0.388	0.00	0.950
MA (mm)	0.09	0.338	0.15	0.160	0.14	0.053
MAAP (mm)	0.13	0.167	0.15	0.146	0.14	0.051
MAML (mm)	–0.05	0.582	0.08	0.422	0.08	0.274
MaxAP (mm)	0.03	0.733	0.18	0.079	0.08	0.244
MaxML (mm)	0.13	0.154	0.14	0.170	0.16	0.026

BMI, body mass index; TUG, Timed Up and Go; TUG cog, Timed Up and Go cognitive; SP, total path length; SPAP, statokinesiogram path length; SPML, statokinesiogram path length; MA: mean COP displacement; MAAP, mean COP displacement from point 0 in the *Y* direction; MAML, mean COP displacement from point 0 in the *X* direction; MaxAP, maximal COP displacement from point 0 in the *Y* direction; MaxML, maximal COP displacement from point 0 in the *X* direction.

**Table 3:** Association between HGS and all outcomes after adjusting for age, sex, and BMI with interaction between sex and HGS.

	*β*	Standard error	*p* value
TUG			
HGS	–0.47	0.13	<0.001
Age	0.28	0.08	<0.001
Sex (M vs W)	–3.12	3.26	0.34
HGS: sex (M vs W)	0.2	0.16	0.203
BMI	–0.03	0.12	0.825
TUG cog			
HGS	–0.49	0.16	0.002
Age	0.30	0.09	0.002
Sex (M vs W)	–1.36	3.97	0.732
HGS: sex (M vs W)	0.14	0.19	0.451
BMI	–0.15	0.14	0.304
Gait speed			
HGS	–0.45	0.14	0.001
Age	0.29	0.08	0.001
Sex (M vs W)	–4.92	3.47	0.158
HGS: sex (M vs W)	0.25	0.17	0.139
BMI	–0.09	0.12	0.482
Chair stand			
HGS	–0.35	0.13	0.010
Age	0.26	0.08	0.002
Sex (M vs W)	–1.68	3.38	0.619
HGS: sex (M vs W)	0.15	0.16	0.343
BMI	–0.06	0.12	0.640
BERG			
HGS	0.53	0.17	0.002
Age	–0.35	0.1	0.001
Sex (M vs W)	–1.54	4.36	0.724
HGS: sex (M vs W)	–0.09	0.21	0.658
BMI	0.05	0.16	0.734
SP-EO			
HGS	–7.54	5.95	0.207
Age	5.14	3.57	0.152
Sex (M vs W)	360.87	151.32	0.018
HGS: sex (M vs W)	–6.63	7.26	0.362
BMI	–8.05	5.43	0.140
SPML-EO			
HGS	–3.66	2.79	0.191
Age	1.19	1.67	0.476
Sex (M vs W)	168.53	70.91	0.018
HGS: sex (M vs W)	–3.38	3.4	0.322
BMI	–7.5	2.54	0.004
MaxML-EC			
HGS	0.13	0.17	0.438
Age	0.11	0.1	0.289
Sex (M vs W)	2.88	4.32	0.506
HGS: sex (M vs W)	–0.15	0.21	0.458
BMI	–0.04	0.15	0.813

HGS, hand grip strength; BMI, Body mass index; TUG, Timed Up and Go; TUG cog, Timed Up and Go cognitive; M, men; W, women; SP, total path length; SPML, statokinesiogram path length; MaxML, maximal COP displacement from point 0 in the *X* direction; EO, eyes open; EC, eyes closed.

## Data Availability

The datasets used and analysed in the current study are available from the corresponding author on reasonable request.
